# *Mustn1*: A Developmentally Regulated Pan-Musculoskeletal Cell Marker and Regulatory Gene

**DOI:** 10.3390/ijms19010206

**Published:** 2018-01-12

**Authors:** Michael Hadjiargyrou

**Affiliations:** Department of Life Sciences, New York Institute of Technology, Old Westbury, NY 11568-8000, USA; mhadji@nyit.edu; Tel.: +1-516-686-7738

**Keywords:** *Mustn1*, musculoskeletal, marker, cartilage, skeletal muscle, bone, tendon

## Abstract

The *Mustn1* gene encodes a small nuclear protein (~9.6 kDa) that does not belong to any known family. Its genomic organization consists of three exons interspersed by two introns and it is highly homologous across vertebrate species. Promoter analyses revealed that its expression is regulated by the AP family of transcription factors, especially c-Fos, Fra-2 and JunD. *Mustn1* is predominantly expressed in the major tissues of the musculoskeletal system: bone, cartilage, skeletal muscle and tendon. Its expression has been associated with normal embryonic development, postnatal growth, exercise, and regeneration of bone and skeletal muscle. Moreover, its expression has also been detected in various musculoskeletal pathologies, including arthritis, Duchenne muscular dystrophy, other skeletal muscle myopathies, clubfoot and diabetes associated muscle pathology. In vitro and in vivo functional perturbation revealed that *Mustn1* is a key regulatory molecule in myogenic and chondrogenic lineages. This comprehensive review summarizes our current knowledge of *Mustn1* and proposes that it is a new developmentally regulated pan-musculoskeletal marker as well as a key regulatory protein for cell differentiation and tissue growth.

## 1. Introduction

Tissues of the musculoskeletal system (bone, cartilage, skeletal muscles, tendons, ligaments) are all comprised of various cell types that exhibit individual differential gene expression patterns. Together with their specific extracellular matrix (ECM), these cells are responsible for the mechanical and functional properties of their respective tissues and organs. Having knowledge of specific genes expressed solely in a given cell type of a particular tissue is not only helpful in identifying and isolating that particular cell type, but more importantly, such genes can be used as markers to track specific lineage specification and differentiation [1], embryonic development [2], and ultimately tissue regeneration [3]. Such individual markers have been identified for musculoskeletal cell types such as osteoblasts, osteoclasts, osteocytes, chondroblasts, chondrocytes, skeletal myoblasts and myocytes, satellite cells, and tenocytes.

As an example, the various stages of osteoblast differentiation can be linked to marker genes to exactly correlate a cellular process, i.e., lineage commitment (i.e., Stro1), proliferation (i.e., *CD44*), maturation (i.e., bone sialoprotein), mineralization (i.e., osteocalcin) cell death (i.e., *Bax*), within a specific cell type such as osteoprogenitor, immature osteoblast, mature osteoblast, and osteocyte [4]. The same is true for other cells types of the musculoskeletal system. Regardless, to date, there is no single marker that is expressed by all musculoskeletal cell types. Such a pan-musculoskeletal cell marker would complement the existing markers and be useful to study multiple cell types simultaneously. *Mustn1*, originally discovered and termed *Mustang* (*mus*culoskeletal *t*emporally *a*ctivated *n*ovel *g*ene) [5], represents such a pan-musculoskeletal cell marker.

The *Mustn1* gene which encodes a 9.6 kDa nuclear protein, was discovered during an expression screen for upregulated genes that play a role in the regeneration of a fractured bone [6]. Bone fracture repair is a complex process that is defined by the interdependent phases of inflammation, angiogenesis, osteogenesis, chondrogenesis, endochondral ossification and remodeling and, thus, it serves as an excellent model of regeneration by enabling the experimenter to isolate individual genes that play a role in one or multiple phases [7]. Following the screen, *Mustn1* was subsequently cloned and its temporal and spatial expression during bone regeneration (following a transverse fracture) was elucidated [5]. Specifically, upregulated *Mustn1* expression during fracture repair was localized to multiple cell types within the callus, including periosteal osteoprogenitors, osteoblasts and proliferating chondrocytes. As *Mustn1* represented a novel gene, its expression was also investigated in multiple adult tissues and it was only found at high levels in skeletal muscle and tendon as well as lower amounts in bone and cartilage, making it a probable musculoskeletal specific gene. This review represents a timely attempt to summarize our knowledge of *Mustn1* in the area of phylogeny, genomic organization, promoter analyses, expression, functional perturbation, and disease states. Hopefully, this review may also induce other researchers to include *Mustn1* as a marker gene given the accrued evidence about its relevance in a substantial number of studies.

## 2. Phylogeny/Genomic Organization

**Figure 1:**
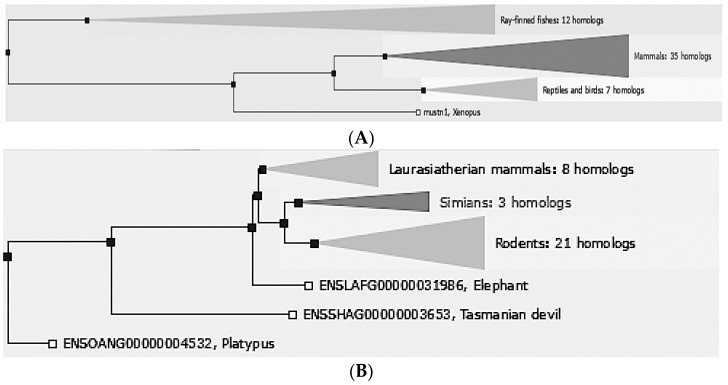
*Mustn1* Phylogeny. Phylogenetic tree displaying evolutionary relationship between *Mustn1* proteins of different vertebrate species. (**A**) All known species. (**B**) Phylogenic relationship of the 35 mammalian homologs.

*Mustn1* is only found in vertebrate organisms, ranging from fish to mammals. Comparative sequence analyses in e!Ensembl [8], revealed that there are 55 different vertebrate homologs: 35 mammals, 7 reptiles and birds, 12 Ray-finned fishes. 1 amphibian (Figure 1A). Further phylogenetic analyses of mammals include closely related laurasiatherian (placental, 8 homologs), simian (apes and monkeys, 3 homologs), and rodents (21 homologs) as well as the more distantly related ones; elephant, Tasmanian devil and platypus (Figure 1B).

**Figure 2:**
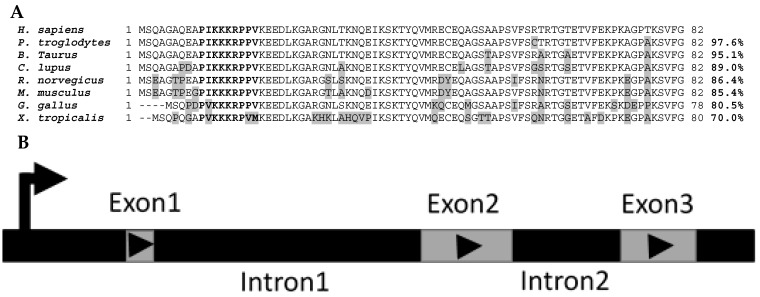
Sequence Homology and Genomic Organization. (**A**) *Mustn1* protein sequences from *H. sapiens* (NP_995325.3), *P. troglodytes* (XP_003950177.1), *B. Taurus* (NP_001035679.1), *C. lupus* (XP_005642307.1), *R. norvegicus* (NP_852033.1), *M. musculus* (NP_852055.1), *G. gallus* (NP_998745.1) and *X. tropicalis* (NP_001165127.1) are aligned and show percent homology (between human and others). Changes in amino acids are shaded in grey. Amino acids not found in some species are denoted by dashes. The NLS, amino acids 10–18, is indicated by bold letters. (**B**) Genomic organization of the *Mustn1* gene showing the 3 exons and 2 introns. Arrow indicates transcriptional start site.

*Mustn1* protein sequence homology between eight species that were identified as putative homologs of one another based on the MUSCLE algorithm [9] and listed in HomoloGene (https://www.ncbi.nlm.nih.gov/homologene/18744, access date: 12 January 2017) clearly shows their homolgy. For example, the *Homo sapiens* protein amino acid sequence is 97.6%, 95.1%, 89.0%, 86.4%, 85.4%, 80.5%, and 70.0% identical to that of *Pan troglodytes*, *Bos taurus*, *Canis lupus*, *Rattus norvegicus*, *Mus musculus*, *Gallus gallus*, *and Xenopus tropicalis*, respectively (Figure 2A). Differences in amino acids between *H. sapiens Mustn1* and those of other species are highlighted in grey (Figure 2A). In bold letters, the highly conserved nuclear localization signal (NLS) located at residues 10–18 is shown among all the species; the top six species show perfect conservation whereas the lower two show a slight divergence in amino acids, due to the overall lower homology (Figure 2A).

Han et al. [10] reported on the porcine *Mustn1* gene which is 78 amino acids and shares 92% and 89% homology with the human and mouse sequence, respectively. More recently, Xu et al. [11] identified the duck *Mustn1* gene and showed that it is comprised of a 78-amino acid sequence with high similarity with that of other birds (96% with zebra finch and 94% with chicken) and lower with mammals (pig (85%), cow (83%), human (83%), rat (86%) and mouse (81%)). Lastly, we recently reported on the cloning of zebrafish *mustn1*. Interestingly, we identified two orthologs, *mustn1*a and *mustn1*b, that shared 71% homology at the amino acid level and whose predicted proteins were highly related to other vertebrate members (63% to human, 61% to frog and chimp, 60% to dog, chicken and cow, 56% to mouse, and 54% to rat) [12]. The discovery and comparative sequence analyses of the vertebrate *Mustn1* gene have given rise to a new protein family, labeled “*Mustang*” (after the original name we assigned to this gene [5]) as outlined by UnitPro (http://www.uniprot.org/uniprot/?query=family:%22MUSTANG+family%22, access date: 12 January 2017).

The genomic organization of *Mustn1* is also conserved within these species. Specifically, *Mustn1* is comprised of 3 exons separated by two internal introns (Figure 2B) in each of these eight vertebrate species shown in Figure 2A. Moreover, in both *H. sapiens* and *P. troglodytes*, the closest of the eight species, *Mustn1* is located on chromosome 3. For the other species, it resides on other chromosomes, again indicating the divergence of this gene in more distantly related species. Interestingly, the genomic organization of the duck *Mustn1* also includes three exons, arranged as those of mammals [11].

## 3. Promoter Analyses

The *Mustn1* promoter element was first isolated, cloned, sequenced and characterized in a myoblast cell line in vitro [13]. The 1512-bp mouse *Mustn1* promoter representing the 5′-flanking region revealed the transcription start site, a TATA box, and multiple putative transcription factor binding sites, particularly AP-1 and AP-2. The activity of this promoter was detected in musculoskeletal cells and exceeded the levels of the control SV40 promoter in C2C12 myoblasts by ~45%. Promoter mutagenesis experiments indicated that one of four AP-1 sites present was required for robust transcriptional activation. The contribution of the AP-2 sites was found to be only marginal in promoter activity. Lastly, we determined that in both proliferating and differentiating C2C12 cells, the immediate early genes, *c-Fos*, *Fra-2* and *JunD* were required for transcriptional activation [13].

**Figure 3:**
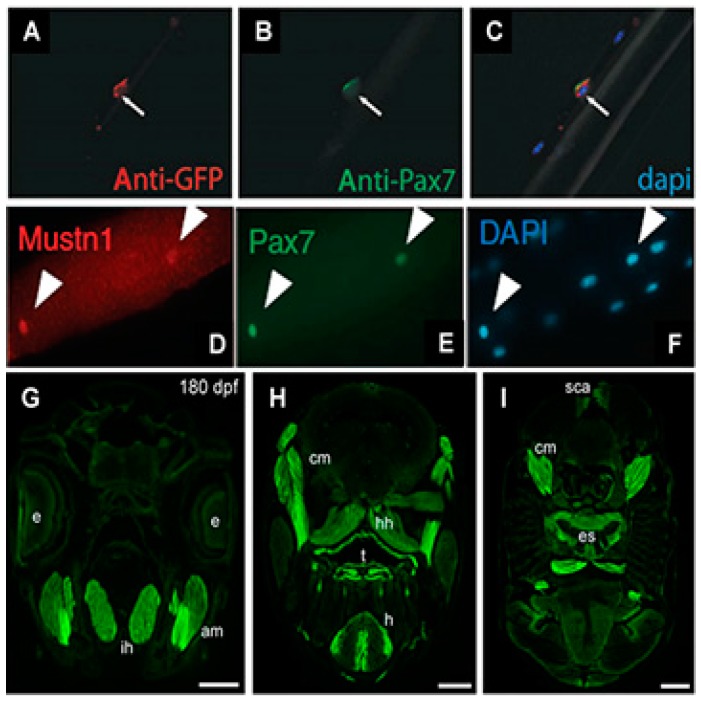
GFP expression as driven by the *Mustn1* promoter. Fixed mouse isolated myofibers co-stained for GFP (**A**), the satellite cell marker Pax7 (**B**) and DAPI (**C**). As can be seen in panel (**C**), there is complete overlay of DAPI, *Mustn1*^PRO^-GFP and *Pax7*. Modified from [14]. Isolated myofibers stained for *Mustn1* (**D**), *Pax7* (E) and DAPI (**F**). Modified from [15]. Transverse adult zebrafish body sections showing *mustn1*b: eGFP expression in jaw, cranial muscles, tongue, heart, and esophagus (**G**–**I**). e, eye; am, adductor mandibularis; ih, interhyal; cm, cranial muscles; hh, hyohyal; t, tongue; h, heart; es, esophagus; sca, supracarinalis anterior. Scale bar = 400 µm. Modified from [16].

Next, we utilized this mouse *Mustn1* promoter to drive the expression of Green Fluorescent Protein (GFP) and generated transgenic mice (*Mustn1*^PR0^-GFP) so that GFP expression would serve as a surrogate for *Mustn1* during skeletal muscle development and regeneration [14]. As we expected based on our previous studies with *Mustn1* expression during development (as described in Section 4 and Section 5), *Mustn1*^PR0^-GFP expression was observed within somites at embryonic day 12 and developing skeletal muscles at embryonic day 15 and 18. Cardiotoxin injury increased GFP expression at 3 days post-injury with decreasing levels observed thereafter. Moreover, GFP expression was detected in newly formed myotubes and satellite cells on freshly isolated, single myofibers which co-localized with *Pax7* (satellite cell marker) expression (Figure 3A–C) [14]. Collectively, these results indicated the expression GFP, as driven by the *Mustn1* promoter, is robust within both developing and regenerating skeletal muscle as well as satellite cells. Consistent with our data, Zhang et al. [15] recently identified *Mustn1* as one of a group of novel regulators of satellite cell homeostasis and also showed that its expression co-localizes with *Pax7* in freshly isolated, single skeletal myofibers (Figure 3D–E).

Very recently, Suarez-Bregua et al. [16] isolated and characterized the zebrafish *mustn1*b promoter by generating transgenic fish with this promoter driving eGFP expression. Specifically, the authors reported stable eGFP expression in a pattern that mirrors that of endogenous *mustn1*b gene expression; in skeletal muscle pioneer cells and somites of embryos and in craniofacial and fin muscles of transgenic larvae. Some eGFP expression was also detected in embryonic cardiac muscle. In the adult fish, eGFP expression was detected in jaw (Figure 3G), cranial muscles, tongue, heart, and esophagus (Figure 3H,I). Light eGFP expression was also detected in the supracarinalis anterior, lateralis superficialis and hypoaxial muscles of the trunk (Figure 3I). Lastly, functional analyses of the *mustn1*b promoter also revealed that the MyoD binding site was crucial for *mustn1*b expression in skeletal muscles [16].

## 4. Skeletal Muscle Expression Analyses

### 4.1. Development

**Figure 4:**
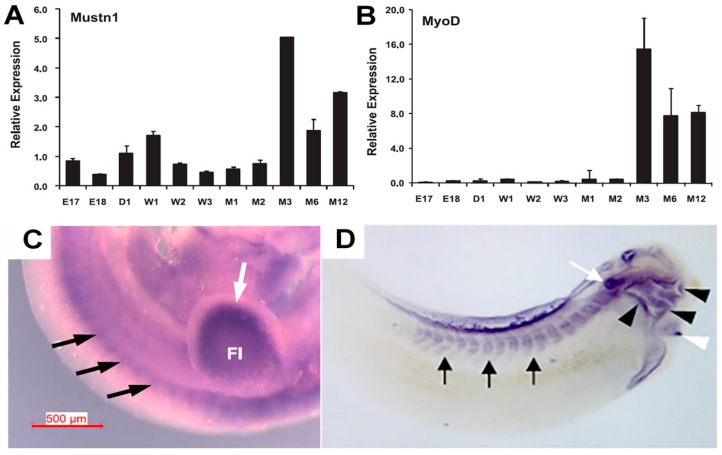
Temporal and spatial expression of *Mustn1* during skeletal muscle development. (**A**,**B**) show Q-PCR analysis of *Mustn1* and *MyoD*, respectively, using RNA isolated from mouse quadriceps from embryonic days 17 and 18 (E17 and E18), newborn day 1 (D1), postnatal weeks 1–3 (W1–W3), postnatal months 1 and 2 (M1, M2), and adult months 3, 6, and 12 (M3, M6, and M12). Modified from [17]. (**C**,**D**) whole mount *Mustn1* in situ hybridization of E10.5 mouse and stage 35 *Xenopus* embryos, respectively. (**C**) Black and white arrows indicate *Mustn1* expression in somites and forelimb, Fl, respectively. Scale bar = 500 µm. Modified from [17]. (**D**) Black arrows denote somites, the black arrowheads denote craniofacial structures, the white arrowhead denotes heart, and the white arrow indicates the otic vesicle. Modified from [19].

*Mustn1* mRNA expression was originally described in the developing mammalian (mouse) skeletal muscle system [17]. Temporal quantitative PCR (Q-PCR) analyses of quadriceps isolated from embryonic day 17 to 12 months of age revealed that *Mustn1* mRNA is expressed at relatively low levels during embryogenesis and up to 2 months of age, but robustly increases at ~5-fold at 3 months of age (a time of increased muscle hypertrophy) and remains at high levels until 12 months (last time point tested) (Figure 4A). This temporal pattern of *Mustn1* mRNA expression mirrored that of the classical myogenesis marker, *MyoD* [18], albeit at lower levels (Figure 4B). Spatially, we also showed abundant *Mustn1* mRNA expression in somites (Figure 4C) and developing skeletal muscles (i.e., trapezius and intercostal), while in adult muscle, *Mustn1* was localized to nuclei at the periphery of myofibers, consistent with location of satellite cells [17], as was also reported by Zhang et al. [15] and shown in Figure 3D. More recently, we described the expression of *Mustn1* during *Xenopus* and zebrafish development. In *Xenopus*, *Mustn1* expression was detected in the paraxial mesoderm and later on in somites and their derived muscle (Figure 4D) [19]. *Mustn1*a and b expression was detected in the somites during zebrafish development as we previously reported [12].

In an experiment comparing the pectoralis major growth rates and muscle mass between control broiler and layer chickens, used as a model for myogenesis, using microarrays, Zheng and colleagues [20], systematically identified differentially expressed genes during different developmental stages, post-hatch day 1, and two, four, six and eight weeks. One of the genes identified in their screen was *Mustn1* and displayed higher mRNA expression in broilers which instead exhibits rapid muscle growth than in layers, especially at two (~3-fold), four (~2.2-fold), six (~2.5-fold) and eight weeks (~1.5-fold), indicating that it plays a role in skeletal muscle hypertrophy. A more recent study sought to identify genes with a key role in determining breast muscle growth by comparing modern pedigree male (PeM) broilers (exhibit rapid growth and muscle development) with a foundation broiler line (Barred Plymouth Rock; BPR) which exhibits slow growth [21]. *Mustn1* was identified as one of the ten most highly upregulated genes (~27-fold) in breast muscle tissue of the modern PeM broiler birds as compared to BPR broilers. As these PeM birds exhibit rapid growth and muscle development, the data suggest, and consistent with the aforementioned studies, that *Mustn1* expression is associated with muscle hypertrophy and has a critical role in myogenesis [21].

Another study with Chinese chickens exclusively focused on the temporal and spatial *Mustn1* mRNA and protein tissue expression in individual skeletal muscles [22]. The authors found that *Mustn1* is predominantly expressed in skeletal muscles, although its expression was also detected in cardiac muscle at various times during development. For example, in pectoralis major and thigh muscles at day 70, *Mustn1* exhibited ~2–3-fold higher increased in mRNA expression as compared to the same muscles from day 1. Moreover, *Mustn1* protein expression analyzed in pectoralis major by Western blotting, also showed increasing concentration at the later time points, as well as in females when compared to male chickens. Lastly, immunohistochemical analyses localized *Mustn1* to peripherally located nuclei in myofibers, presumably staining satellite cells, not surprising given the results shown in Figure 3. Based on these data, the authors concluded that *Mustn1* mRNA/protein expression in chickens is most abundant in skeletal muscle and it is differentially regulated during post-hatch muscle growth and corroborating its role in muscle development [22].

Pectoralis major growth is also used as a model for studying myogenesis in birds and Xu and colleagues [23] used Peking ducks to identify genes expressed during its development [23]. Specifically, they found that *Mustn1* mRNA expression is temporally regulated during the first 8 weeks of postnatal development; increased from week 2 (~2.5-fold) to 8 (~3.5-fold) with the highest significant peak of expression at week 6 (~13-fold). Consistent with the aforementioned chicken studies, the authors concluded that *Mustn1* is associated with the rapid development of breast muscle in Peking ducks [23]. Another model of skeletal muscle hypertrophy is represented by the callipyge mutation in sheep. This mutation results in postnatal skeletal muscle hypertrophy characterized by an increase in type IIb fibers (fast-twitch glycolytic); in this model, it was shown that *Mustn1* expression was downregulated at 12 weeks of age in the affected sheep as were troponin T1 and troponin C [24]. These findings seemed consistent with the association of troponin T1 and troponin C with slow type 1 muscle fibers. Perhaps *Mustn1* may also play a bigger role in slow vs. fast twitch fibers but that remains to be determined, although some peripheral evidence does exist and is discussed in the next Section 4.2.

A comparison of gene expression of longissimus dorsi between two strains of pigs, the Korean native pig, which is characterized by relatively high intramuscular fat content, with that of Yorkshire, a western breed that grows faster and contains more lean muscle was also conducted [25]. *Mustn1* was found to be one of 14 genes related to cell proliferation and differentiation that was downregulated in the skeletal muscle of the Korean native pig as defined by a greater than a 1.5-fold change in expression. These microarray data were also verified by Q-PCR and showed an ~3-fold decrease in *Mustn1* expression. In addition, 7 other genes with functions in the structural component of skeletal muscle were also downregulated [25].

### 4.2. Exercise

An interesting set of studies focused on the differential expression of muscle genes during exercise and reported differential *Mustn1* expression. In a human study, Kostek et al. [26], compared changes in gene expression within 24 h of an acute bout of resistance training using lengthening (eccentric) contractions, which induce greater increases in muscle size and shortening (concentric) contractions, conducted simultaneously in the quadriceps of different legs. *Mustn1* mRNA expression was found to steadily increase over the 24 h of eccentric contractions while it remained essentially unchanged during concentric contractions. Within the four time points assayed (0, 4, 6, and 24 h of exercise), *Mustn1* expression was significantly upregulated with an ~2.9 and ~6-fold at 6 h and 24 h, respectively, for eccentric as compared to concentric contractions suggesting that *Mustn1* may be involved in the anabolic responses of muscle to increased physical activity [26].

Oh [27] performed an experiment in a rat model to determine if resistance exercise affected *Mustn1* expression. Thirty-two male rats were equally separated into sedentary (control group) and exercise-trained groups (resistance). The rats in the resistance exercise group were trained to climb an 85-degree incline ladder with weights secured to their tail (10 times a day, 3 days per week) for 8 weeks. At 4 and 8 weeks of exercise, the flexor halucis longus was harvested and analyzed for *Mustn1* expression. At both 4 and 8 weeks of resistance exercise, *Mustn1* mRNA expression increased significantly (~3.7-fold and ~2.1-fold, respectively) in the exercised muscle as compared to controls suggesting that *Mustn1* had a positive effect on myogenesis during resistance ladder exercise [27]. The author also performed a similar exercise (same regimen) study that utilized microarrays to identify differential gene expression and found *Mustn1* to be upregulated (~3- and ~1.5-fold as verified by Q-PCR) again after four weeks and eight weeks, respectively, of exercise training and indicated that *Mustn1* is potentially an important gene involved in resistance exercise and muscle hypertrophy [28]. McKenzie et al. (2011) [29] also used rats to determine differential gene expression involved in the “stress” response of the gastrocnemius (fast twitch) and soleus (slow twitch) muscles after a single aerobic exercise bout (run for 2 h at 20 m·min^−1^). In contrast to the gastrocnemius where *Mustn1* expression was not detected, in the soleus, it was upregulated (~4-fold vs. sedentary control) as detected by microarray. While the authors did not verify the differential expression of *Mustn1* via Q-PCR, the data supports the notion that *Mustn1* is an early gene response gene to an anabolic stimulus, at least in slow twitch skeletal muscle fibers [29].

A final exercise study investigated gene expression profiling of porcine skeletal muscle in the early recovery phase following acute physical activity [30]. The exercise regimen consisted of a treadmill with a stepwise increasing speed from 0.4 to 5.2 km/h in increments of 0.4 km/h every 2 min until for approximately 30 min. The biceps femoris and longissimus dorsi were harvested immediately after exercise (T0), one hour after (T1), and three hours (T3) and compared to unexercised controls. *Mustn1* expression was significantly upregulated ~2.6-fold at T3 in the biceps femoris but its expression remained unchanged in longissimus dorsi (as assayed initially by microarray and verified by Q-PCR). Further, *Mustn1* expression was also detected in isolated satellite cells from the vastus intermedius muscles that were used in proliferation and differentiation assays. Specifically, *Mustn1* revealed a statistically significant higher level of expression (~2-fold) during differentiation of myoblasts into myotubes in comparison to proliferating cells. This is consistent with our data showing the involvement of *Mustn1* in myofusion and formation of myotubes [17] (discussed in Section 7). Lastly, the authors hypothesized that the applied intensive physical exercise likely activated resident satellite cells (which do express *Mustn1*, [14,15] and Figure 3) in order to repair muscle injuries and to prepare for another bout of exercise [30].

## 5. Cartilage Expression Analyses

**Figure 5:**
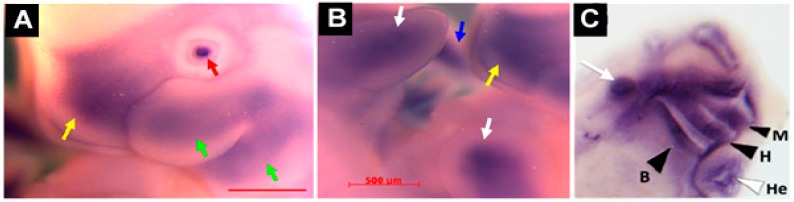
Spatial expression of *Mustn1* during skeletal development. (**A**,**B**) whole mount *Mustn1* in situ hybridization of E11.5 mouse embryos showing distinct staining in branchial arches (green arrows), frontonasal process (yellow arrows), and fore and hind limbs (white arrows), lens (red arrow) and posterior tail bud (blue arrow). Scale bar = 500 µm. Modified from [31]. (**C**) whole mount *Mustn1* in situ hybridization of stage 39 *X. laevis* showing staining in craniofacial structures as shown by the black arrowheads (M, mandibular arch; H, hyoid arch; B, branchial arch); the white arrowhead denotes heart, and the white arrow indicates the otic vesicle. Modified from [19].

As previously mentioned, upregulated *Mustn1* expression during bone regeneration was localized to multiple cell types within the fracture callus, including proliferating chondrocytes [5]. More recently, we also detected *Mustn1* expression in articular cartilage chondrocytes as well as those in the germinal/reserve zone of the growth plate (unpublished observations). During mouse embryonic development, *Mustn1* expression was detected as early as 10.5 days post conception (dpc) in several areas of active cartilage and bone formation [31]. For example, robust expression was present in the craniofacial region, especially the developing first branchial arch that begins to divide into the maxillary and mandibular components (Figure 5A). In addition, hybridization was also detected in the frontonasal process (Figure 5A). Similarly, the fore and hind limb buds also displayed robust *Mustn1* expression at 10.5 dpc and as development proceeded to 11.5 dpc, staining was again present along the entire length of both fore and hind limb buds and the posterior tip of the tail (Figure 5B).

This is consistent with our findings in a rat model, where 16 dpc embryos showed *Mustn1* expression in mesenchymal condensations in developing digits and intervertebral discs, as well as the in the perichondrium of developing vertebral bodies [5]. Similarly, we also observed intense *Mustn1* expression in the cranial region during *Xenopus* development, specifically, at late tadpole stage 35, in anterior structures corresponding to mandibular, hyoid, branchial and other head cartilaginous tissues [19] (Figure 5C). In Zebrafish, *mustn1*b mRNA was primary detected in the pharyngeal arches at 72 h post fertilization (hpf) and by five days, where the pharyngeal arch mesoderm begins to differentiate into the cartilage structures that will eventually form the jaw [32], its expression was present in the ceratohyal and ceratobranchial elements of the pharyngeal skeleton. This pattern of expression was most likely a progression from the pharyngeal arch expression detected at 72 hpf [12] consistent with the data using transgenic fish (*mustn1*b: eGFP) (Figure 3) [16].

An interesting study was performed in rats in order to identify differentially expressed genes in mandibular condylar cartilage during natural growth and under mechanical strain as a result of mandibular advancement [33]. To induce mandibular advancement, acrylic bite-jumping appliances were fitted to the upper incisors of the experimental rats to produce a continuous 3.5 mm anterior and 3mm inferior displacement of the mandible. The appliances were kept cemented in place and groups of rats were sacrificed on days 1, 3, 7, 9, 14, 21, 30, and 33 and compared to control (no appliances). *Mustn1* was one of five genes that were upregulated in the experimental rats and involved with different stages of chondrogenesis in mandibular condyle growth. Although *Mustn1* expression did not change in the control animals (natural growth), its expression was found at all days tested (1, 7, 9, 14, 30, 33), but was only significantly upregulated at days 7 (~2-fold), 9 (~2-fold), 14 (~3-fold), and 21 (~2-fold) during the advancement of the mandibular condyle in the experimental group, suggesting a role in the activation of mandibular condylar cartilage formation [33].

More recently, the effect of pulsed and continuous ultrasound (US) exposure on chondrogenesis-related gene expression was tested in rat tibial articular cartilage [34]; pulsed US is used clinically to accelerate fracture healing [35,36]. Three groups of rats were tested, a control group which was treated with sham sonication, a pulsed US group that received a pulse rate of 20%, at a frequency of 1 MHz, and an intensity of 1.5 W/cm^2^ for 10 min and a continuous US group that was exposed continuously at a frequency of 1 MHz and an intensity of 1.5 W/cm^2^ for 10 min. Each group received a single US treatment exposure. The two genes that were selected to monitor chondrogenesis were *Mustn1* and the classical chondrogenic marker, Sox9, a master regulatory gene encoding for a transcription factor [37]. Results showed that mRNA expression of both *Mustn1* and Sox9, increased significantly in the continuous (~24% and 37%, respectively) and pulsed groups (44% and 52%, respectively), but the increase of *Mustn1* mRNA in the continuous US group was significantly more prominent than in the pulsed group (22% and 15%, respectively). Based on these results, the authors suggest that US stimulates chondrogenic gene expression in articular cartilage and may potentially serve as a therapeutic modality [36].

## 6. Bone and Tendon Expression Analyses

**Figure 6:**
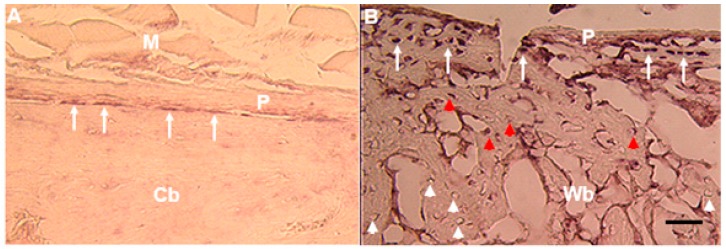
*Mustn1* expression in bone. In situ hybridization of *Mustn1* in sections obtained from intact bone (**A**) and post-facture day 5 callus (**B**). White arrows indicate *Mustn1* expression in periosteal osteoprogenitors of intact bone (**A**) and young osteoblasts in a post-fracture day 5 callus (**B**). Red arrowheads indicate the expression of *Mustn1* in trapped osteoblasts (**B**); red and white arrowheads show the gradual decrease and absence, respectively, of *Mustn1* expression. Cb, cortical bone, M, muscle, P, periosteum, Wb, woven bone. Scale bar = 50 µm. Adapted from [5].

In adult bone, *Mustn1* expression is localized to periosteal osteoprogenitor cells (Figure 6A) and during fracture repair its expression is detected in osteoblasts as well osteocytes [5,38] (Figure 6B). Similarly, *Mustn1* was also identified by microarray analysis in a fracture repair study utilizing the same transverse femoral fracture model as in our study, as the gene with the greatest fold increase from exposure to alcohol consumption as compared to control (no alcohol) at post-fracture day 3 [39]. The upregulated expression of *Mustn1* was also verified by Q-PCR (~3-fold) and it coincided with the upregulated expression of Testin (~2-fold), a gene previously identified in the mouse to be expressed at embryonic day 10.5 in the mesenchyme of all branchial arches and also in the frontonasal processes, as well as in mesenchyme of the limb buds [40]. Incidentally, Testin’s pattern of embryonic expression mirrors that of *Mustn1*, as we previously described in the mouse [31].

A well-designed study examined differential gene expression between pre-osteoblasts, osteoblasts and osteocytes using visual markers of bone lineage cells derived from dual GFP reporter mice. In these mice osteocytes expressed GFP (topaz) directed by the DMP1 promoter, while pre-osteoblasts and osteoblasts expressed GFP (cyan) expression driven by 2.3 kb of the Col1a1 promoter [41]. Using this innovative approach, *Mustn1* was identified as a gene expressed by both Col2.3cyan+ (osteoblasts), and DMP1topaz+ (preosteocytes and osteocytes), again consistent with our previous observations [5,38].

The expression of *Mustn1* was also detected in an experiment where cultured primary osteoblasts, from calvarial and trabecular bone, isolated from *PTHrP*+/+ and −/− mice were exposed to 1 g or simulated microgravity for 6 days with or without intermittent (2hr daily) PTHrP1–36 treatment [42]. *Mustn1* expression was upregulated ~2.2-fold in *PTHrP*+/+ osteoblasts exposed to simulated microgravity as well as ~3.9-fold in *PTHrP*−/− osteoblasts at 1 g. Additionally, it was found that *Mustn1* expression was down-regulated by ~0.6-fold in osteoblasts treated with PTHrP. *Mustn1* was also one of only 24 genes whose expression was common to all three conditions (up-regulated in simulated microgravity and *PTHrP* ablation and down-regulated by PTHrP1–36 treatment). Lastly, and more importantly, cluster analysis of genes whose expression was modified by microgravity and similarly affected by *PTHrP* ablation placed *Mustn1* in the same cluster as genes involved in bone growth, mineralization and bone morphogenetic protein (BMP) metabolism, suggesting a role of *Mustn1* in these key osteogenic processes [42].

Although we previously demonstrated that *Mustn1* is expressed in tendon [5], the only study that provided additional evidence that *Mustn1* is expressed in isolated tenocytes was reported recently from Mueller and colleagues [43]. In this study, the authors isolated tenocytes cells from tail, Achilles, and hind limb deep digital flexor tendons from three-month-old male rats and cultured these into a monolayer as well as fibrin gels, respectively, for 7–10 days. RNA from both native tendons as well as from the three-dimensional tenocyte cultures were then used for gene expression profiling analyses. Results revealed that *Mustn1*, together with other well-known tendon genes such as tenomodulin, elastin, keratocan, and lubricin, were more highly expressed (~3-fold) in native tendon than in monolayer or the three-dimensional tenocyte cultures. The data from all expression studies described in Section 4, Section 5 and Section 6 are summarized in Table 1.

## 7. Functional Perturbation and Regulation

The initial functional indication for *Mustn1* was derived from its amino acid sequence that reveled a NLS, thus making it a probable nuclear protein. We subsequently verified its subcellular localization by transfection experiments using a GFP-*Mustn1* fusion protein that labeled only the nuclei green, indicating active translation and nuclear import [5]. The NLS was also demonstrated to be functional through nuclear localization of zebrafish *mustn1*a fused to GFP [44]. It is of interest to note that in our study as well as that of Cholski et al. [44], nucleoli and the nuclear envelope were devoid of any staining suggesting that *Mustn1* is not involved in associated “housekeeping” processes (i.e., rRNA synthesis; nuclear import/export and/or structural functions). Although, these experiments suggested a nuclear function for *Mustn1*, they did not address its function directly, but we hypothesized that *Mustn1* may function as a co-activator or co-regulator of transcription as part of a larger multi-protein transcription initiation complex [5,31].

**Figure 7:**
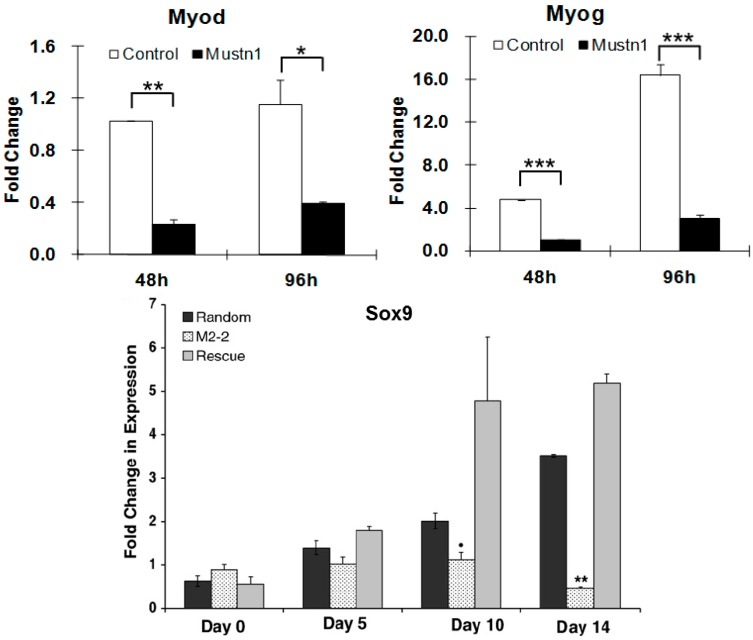
*Mustn1* silencing leads to downregulation of myogenic and chondrogenic transcription factor mRNA. Top graphs: RNA from control (GFP) and *Mustn1* RNAi-treated C2C12 cells was isolated at 48 and 96 h after plating and subjected to Q-PCR to assay *Myod* and *Myog* expression. * *p* < 0.05, ** *p* < 0.01, *** *p* < 0.001, determined by one-way ANOVA with Tukey’s post hoc test. Adapted from [17]. Bottom graph: Confluent RCJ cells were stimulated to differentiate at Day 0 and mRNA was isolated and assayed via Q-PCR at Days 0, 5, 10 and 14 for *Sox9*. Random = random RNAi treated cells; M2-2 = *Mustn1* RNAi treated cells; Rescue = transiently transfected M2-2 cells with a *Mustn1* expression vector. Significance was determined by Mann–Whitney test vs. random Expression levels. ** *p* <0.001, ● *p* <0.01. Adapted from [31].

To directly address the function of *Mustn1* in cells of the musculoskeletal system, myogenic and chondrogenic, we utilized the in vitro and in vivo approach of overexpression and silencing [17]. Using the C2C12 pre-myoblastic cell line as a model for myogenic differentiation, we showed that silencing of *Mustn1* mRNA via RNA interference (RNAi) had no effect on the proliferation of these cells. In contrast, *Mustn1* silencing significantly impaired myoblast differentiation, preventing myofusion and ultimately myotube formation. Moreover, *Mustn1*-silenced myoblasts elongated poorly and were mono-nucleated as opposed to large, multi-nucleated myotubes present in the control cells, even after 6 days in the presence of myogenic differentiation medium. Additional immunocytochemical analyses of *Mustn1*-silenced cells demonstrated significant reductions in both, the amount and timing of expression of the myogenic markers, myogenin (*Myog*) and myosin heavy chain (*Myhc*) at 4 and 6 days of differentiation. These decreases in *Myog* and *Myhc* protein expression in *Mustn1*-silenced cells were also associated with robust decreases in *Myog*, *MyoD* (Figure 7), *Myhc* and desmin (*Des*) mRNA expression, as well as those of myofusion markers, Calpain1 (*Capn1*), Caveolin 3 (*Cav3*), and Cadherin 15 (M-cadherin; *Cadh15*). Taken together, the data indicates that *Mustn1* is an essential regulator of myogenic differentiation, myofusion and myotube formation [17]. A more recent study also showed that knockdown of *Mustn1* via RNAi inhibited expansion of mouse primary skeletal muscle stem cells [15].

Similar experiments were conducted with the pre-chondrocytic RCJ3.1C5.18 (RCJ) cell line [31]. This cell line represents a heterogeneous cell population capable of differentiation from proliferating chondrocytes to terminally differentiated hypertrophic chondrocytes [45]. We showed that *Mustn1* overexpression (~2–6-fold) had no statistically significant changes in either proliferation or chondrogenic differentiation. In contrast, both proliferation and differentiation (as assayed by proteoglycan production and cartilage specific gene expression) were significantly reduced in the *Mustn1* silenced cell lines. Specifically, *Mustn1* silencing led to an ~55–75% reduction in cell number. Similarly, an ~34–40% reduction in proteoglycans was observed as compared to parental and random control lines, which was also accompanied by significant downregulation of mRNA levels of the chondrogenic specific marker genes, *Sox9* (Figure 7), Collagen type II (*Col II*), and Collagen type X (*Col X*), indicating that *Mustn1* is a necessary regulator of chondrocyte function [31].

**Figure 8:**
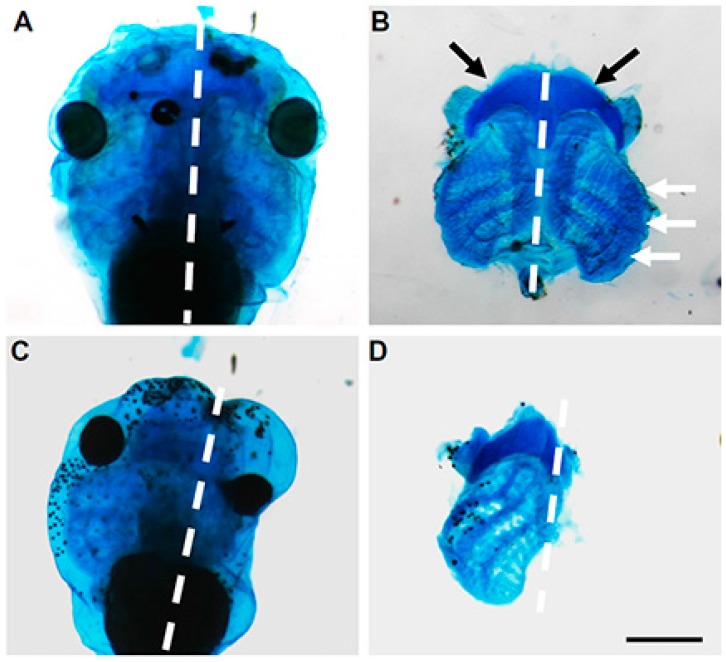
Effect of *Mustn1* knockdown on cartilage development. Ventral views of unilaterally injected *Xenopus* embryos (stage 45) following Alcian blue staining. Control morpholino-injected embryo, with a dotted line indicating the midline (**A**). The same embryo shown in (**B**) after the removal of tissue. (**C**,**D**) show a representative control and *Mustn1* morpholino-injected embryo, respectively, with alteration of midline and loss of symmetrical cartilaginous structures on injected side. Black arrows indicate the ceratohyal cartilage; and white arrows denote the gill (branchial) arches. Scale bar = 0.5 mm. Adapted from [19].

These results were further supported by in vivo experiments in *Xenopus* where we utilized antisense morpholinos to downregulate *Mustn1* at the 4-cell stage [19]. Specifically, the antisense morpholinos were injected into the dorsal and anterior tissues of the developing embryo, including the head and anterior paraxial mesoderm, as well as the anterior neural-ectodermal margin from which the cranial neural crest cells (NCCs) originate. Targeted knockdown of *Mustn1* resulted in phenotypes characterized by small or absent eyes (68% of injected tadpoles), a shortened body axis (49%), and tail kinks (45%) at both stages 37–38 and 40 that corresponded to early swimming/feeding tadpoles. Additionally, when we unilaterally injected the same *Mustn1* antisense morpholinos, we observed the same gross morphological defects on the injected side. More importantly, *Mustn1* knockdown reduced cranial *Sox9* mRNA expression and showed dramatic disruption in ~93% of the examined tadpoles that showed disrupted cartilage formation as detected by Alcian blue staining and in some cases, a complete absence of cartilaginous structures associated with the eye, as well as the ceratohyal cartilage and gill arches [19] (Figure 8). Such failure of cranial embryonic cartilage development observed with *Mustn1* downregulation, is consistent with *Xenopus* phenotypes resulting from knockdown of other known chondrogenic regulators such as *Sox9* (depletion shows reduction in eye size and anterior Alcian blue-positive cartilaginous matrix, [46]) and *Runx2* (ablation of cranial cartilage formation, [47]).

Zebrafish *mustn1*a was found as a gene whose expression was induced (~30-fold) by *Foxj1*, a winged-helix transcription factor that serves as the master regulator of motile cilia biogenesis, through a systematic effort (microarrays and functional genomics) to discover novel ciliary genes [44]. In the same study, morpholino knockdown of zebrafish *mustn1*a resulted in a curved body axis, similar to what we observed with *Mustn1* knockdown in *Xenopus* [10] as described above. Lastly, zebrafish *mustn1*a knockdown also caused defects in otolith and left-right asymmetry, as well as curling of cilia and disorganized γ-tubulin expression, a marker of the basal bodies [44]. The data from all expression studies described in this section are summarized in Table 2.

The observation that *Mustn1* knockdown both in vitro and in vivo, resulted in the downregulation of the master regulatory transcription factors, *MyoD*, *Myog* and *Sox9*, suggest that *Mustn1* is involved in early stages during myogenic and chondrogenic differentiation, and at least in vivo, provides the first direct evidence that *Mustn1* is required for formation of embryonic cartilage. This is not surprising given that *Mustn1* expression is robust in these tissues during embryonic development (i.e., somites and limb buds), as previously reported [5,17,19,31] and outlined in Section 4 and Section 5. More importantly, these data also raise the question of whether *Mustn1* is a direct transcriptional regulator of *Myod*, *Myog* and *Sox9* expression. As *Mustn1* lacks a DNA binding motif, it precludes it from being a direct transcription factor and thus further supports the notion that *Mustn1* probably functions as a musculoskeletal co-activator or co-regulator. Specifically, we speculate that *Mustn1* is part of a transcriptional initiation complex responsible for the activation of these master regulatory genes during both myogenesis and chondrogenesis.

Although these data implicate *Mustn1* in the activation of *Myod*, *Myog* and *Sox9*, they do not indicate which signaling pathway is involved with *Mustn1* activation. However, prior research into several important musculoskeletal signaling pathways implicates them in regulating *Mustn1* transcription. One such study involves Shh, Sonic hedgehog, which is a member of the well-known embryonic Hedgehog signaling pathway [48]. It is well established that the downstream target genes regulated by morphogens such as Shh and other hedgehog proteins, Desert Hedgehog (Dhh) and Indian Hedgehog (Ihh), are ultimately responsible for processes such as cell proliferation, differentiation and skeletal development [49]. For example, a murine multipotent mesodermal cell line (C3H10T1/2) was used to elucidate transcriptional targets of Shh. Following overexpression of Shh in C3H10T1/2 and a microarray screen, *Mustn1* was one of 141 genes showing a >1.5-fold increase in expression. Some of the additional genes identified include other transcriptional regulators, as well as those involved in developmental processes, including cellular proliferation and differentiation [50].

C3H10T1/2 cells were also utilized as a model of osteoblastic differentiation in order to discover transcriptional targets of the Wnt signaling pathway, particularly, Wnt3A [51]. Activation of signaling pathways by Wnts ultimately leads into a wide array of developmental processes that include cell proliferation, migration, differentiation, establishment of cell polarity, and specification of cell fate [52]. Results from this experiment identified *Mustn1* as a Wnt3A signaling target gene, with an ~2–3-fold increase in its expression. Interestingly, many of the genes that showed Wnt3A-stimulated expression induction were previously identified as Wnt3A targets, which validates the authors’ experimental approach. Further, a subset of these Wnt3A target genes are already known to play a role in osteoblast function and include *Axin2*, *Bmp4*, *Cyr61*, *Ctgf*, *Hes1*, *Igfbp2*, *Omd*, *Tgfb3*, *Thbs1*, *Twist1*, and *Wisp1* [51]. These results suggest that *Mustn1* expression in osteoblasts may be regulated by Wnt signaling. As such, *Mustn1* (along with other well-established genes) appears to be a target of multiple signaling pathways, including Hedgehog and Wnt, reinforcing the fact that *Mustn1* has a critical role in cellular processes that lead to the development of the various tissues of the musculoskeletal system.

## 8. Disease States

Beyond understanding the basics aspects of *Mustn1* biology (i.e., cloning, genomic structure, promoter analysis, expression, functional perturbations, etc.), we also want to investigate whether it is associated with any particular disorders or disease states. There are now several studies in the literature that have identified *Mustn1* in various disease states of the musculoskeletal system. A number of human genome-wide association studies (GWAS) searching for risk alleles for osteoarthritis, a musculoskeletal disease characterized by gradual loss of articular cartilage accompanied with physiological alterations in the subchondral bone and the synovium, have been conducted. Reynard and Loughlin [53] reviewed a number of GWAS studies on osteoarthritis and summarized the data showing a number of potential risk genes for the disease. In addition to highly plausible candidate genes such as *RUNX2* and *CHST11*, a transcription factor active in joint development and an enzyme that adds sulfate groups to cartilage proteoglycan, respectively, one of the studies reviewed (arcOGEN, with individuals of European and North American of European descent) also identified *Mustn1* as one of the signals. This led the authors to suggest that the arcOGEN study has provided very novel insights into the etiology of osteoarthritis by the fact that the majority of the genes identified, including *Mustn1*, have not previously been suggested to have a role in osteoarthritis [53]. The plausibility that *Mustn1* is linked to osteoarthritis is not surprising as *Mustn1* is known to be expressed in adult articular cartilage, especially by proliferating chondrocytes in the superficial/tangential zone (unpublished observations).

Aside from cartilage, *Mustn1* expression was also identified in skeletal muscle diseases. Van Lunteren and Moyer [54], conducted an experiment searching for differentially expressed genes in the diaphragm muscle of streptozotocin-induced diabetic rats using microarrays. Data showed that 105 genes with at least 2-fold significantly changed expression (55 increased and 50 decreased) in the diaphragm of the diabetic rats. *Mustn1* was found to be one of the genes whose expression increased by ~3.2-fold in the diabetic diaphragm and following ontological analyses it was assigned to a group with nine other upregulated genes that are known to be involved in the formation and organization of tissue and organ structure (morphogenesis and organogenesis) [54].

Another experimental study examined differential gene expression in broiler chickens that suffer from a muscle disorder characterized by palpably “hard” or tough breast muscle (referred to as “Wooden Breast”) [55]. This myopathy predominantly affects the pectoralis major, and occasionally minor muscles and is associated with multifocal degeneration and necrosis of the muscle tissue with infiltration of inflammatory cells. Results from this study showed that *Mustn1* was upregulated ~4.9-fold in the affected birds and the authors speculate that this indicates compensatory hypertrophy or muscle repair secondary to muscle damage [55]. Again, not surprising since *Mustn1* is expressed during skeletal muscle hypertrophy [17,24] and regeneration [14].

Kennedy’s disease/Spinobulbar muscular atrophy (KD/SBMA) is a degenerative neuromuscular disease that affects males and is caused by polyglutamine expansion mutations of the androgen receptor (*AR*) gene. Halievski and colleagues [56] used a transgenic mouse model of KD/SBMA because it overexpresses wild-type *AR* exclusively in myocytes and has a severe phenotype following acute androgen treatment in females, which reproduces the sex limited (male) and androgen dependent features of the KD/SBMA phenotype [57]. Thus, treating non-symptomatic females with testosterone induces disease symptoms within 3 days and by 7 days these female mice develop severe symptoms that are typically seen in diseased males. Using microarray analysis of RNA from muscles from both transgenic females (treated for three or seven days with testosterone), it was shown that *Mustn1* expression increased in KD/SBMA muscles of treated female (~11-fold) and affected males (~3-fold), leading the authors to suggest that since *Mustn1* expression is found during skeletal muscle regeneration [14], hypertrophy [17,24], and exercise [26,27,28,29,30], its increase in KD/SBMA mice may contribute to their ability to recover following testosterone removal [56].

Duchenne muscular dystrophy (DMD) is an inherited X-linked lethal muscle wasting disease caused by a mutation in the dystrophin gene that normally encodes for a protein that links the muscle cytoskeleton through a membrane complex to the extracellular matrix. The absence of dystrophin causes various structural and signaling defects in muscle, leading to dystrophic myofibers that are susceptible to damage during mechanical contractions. A recent study with dogs, investigated whether systemic delivery of skeletal muscle-resident stem (MuStem) cells isolated from a 10-week-old healthy dog could serve as a therapeutic modality for the treatment of the Golden Retriever muscular dystrophy (GRMD) dog model, which is characterized by rapid progressive clinical dysfunction and severe muscle tissue remodeling [58]. These MuStem cells are early myogenic progenitors and uncommitted cells that can be induced to differentiate into myogenic cells. Specifically, the authors compared global gene expression profile in biceps femoris between healthy, GRMD and MuStem cell treated GRMD dogs four months after allogenic MuStem cell transplantation. Results showed that *Mustn1* was one of sixteen genes with significant upregulated expression (~2.5-fold as verified by Q-PCR) in MuStem GRMD dog muscle as compared to untreated control. Some of these 16 genes are also involved in processes such as muscle regeneration, cellular homeostasis, and metabolism. The authors concluded that their results clearly indicate that MuStem cells can positively affect many biological processes, even several months after their transplantation leading to an improvement in the treated GRMD dogs. Moreover, it is the actual gene expression that afforded the treated GRMD dogs the ability to maintain robust muscle fiber regeneration activity that probably led to the stability of the dystrophic muscles [58].

Clubfoot is a malalignment of the bones and joints of the foot and ankle, and affects 1 in 1000 live births, however, little is known about its genetic or developmental basis. A missense mutation in the Pitx1, a bicoid homeodomain transcription factor, was previously identified in humans with a spectrum of lower extremity abnormalities, including clubfoot [59]. Because this mutation reduces Pitx1 activity, the authors hypothesized that *Pitx1* haploinsufficiency could also cause clubfoot. Thus, *Pitx1*^+/−^ mice were generated and showed a clubfoot-like phenotype associated with deficits in vasculature and bone and muscle volume in the affected limbs [60]. These observed morphological abnormalities suggested that the clubfoot phenotype results from changes during early embryonic limb development that affect all tissues in the limb. As such, *Pitx*^−/−^ mice were generated and skeletal muscle gene expression was analyzed via microarray using E12.5 hindlimb buds and compared to those of wild-type mice. Interestingly, *Mustn1* was one of 19 genes related to muscle development whose expression was downregulated (~2.3-fold) in the E12.5 hindlimb buds from the *Pitx*^−/−^ mice indicating that the muscle hypoplasia observed was due to abnormal early skeletal muscle development [60].

## 9. Conclusions

**Figure 9:**
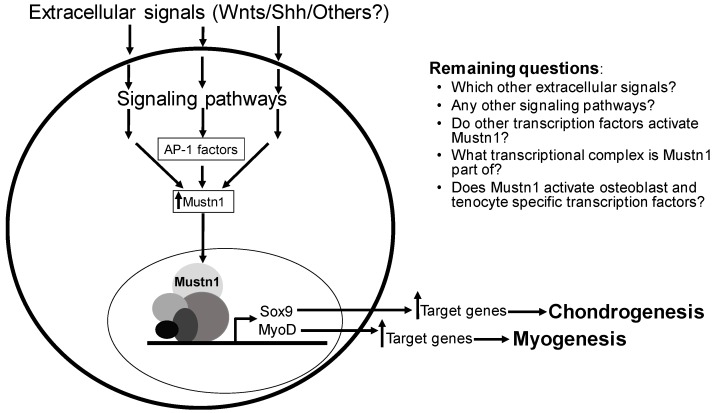
A schematic representation of *Mustn1* regulation and function. The regulation and function of *Mustn1* in tissues of the musculoskeletal system such as cartilage and skeletal muscle as proposed in this schematic is experimentally supported. Whether the same is true for bone and tendon remains to be determined. Moreover, many mechanistic questions still remain unanswered.

It is abundantly clear that since our initial report on the cloning and expression studies of *Mustn1* [5], a large number of studies implicate its expression with a role predominantly in tissues of the musculoskeletal system, as outlined herein. This was further demonstrated by the *Mustn1*-specific functional perturbation studies described in Section 7. And more recently, *Mustn1* expression has been linked to various disease states related to the musculoskeletal system as described above. Despite the wealth of *Mustn1*-related information, there is still much that we do not know about this gene. For example, no one has conducted a general or conditional knockout (the gold standard of determining gene function) of *Mustn1* to show the consequences of its ablation on the developing musculoskeletal system. My laboratory is now in the process of generating a *Mustn1* conditional knockout in cartilage and we are hopeful that we will observe an interesting phenotype given the aforementioned functional knockdown data, both in vitro and in vivo, that clearly showed impairment of chondrogenic differentiation [31] and cartilage formation in general [19]. Moreover, generating a *Mustn1* specific knockout in bone, skeletal muscle, and tendon will also be an interesting avenue of research in order to be able to compare the outcome of these studies between all of the major tissues of the musculoskeletal system. In addition to directly determining *Mustn1* function, we also need to increase our knowledge of how its expression is regulated; by what specific signaling molecules and which signaling pathways (Figure 9). Along with this, we should also seek to decipher which other transcription factor(s) are responsible for its direct expression.

Lastly, and more importantly, since we hypothesize that *Mustn1* functions as a cofactor of a transcriptional initiation complex, it would be a worthwhile effort to identify its interacting proteins. To this end, a study focusing on LNX1, Ligand of Numb, protein X 1, a RING (Really Interesting New Gene) domain-containing E3 ubiquitin ligase identified *Mustn1* as one of 62 potential interacting proteins [61]. This search was based on the presence of PDZ (Post-synaptic density, 95 kDa, Discs large, Zona Occludens-1) domains, which are protein interaction domains that bind to the carboxy-terminal amino acids of binding partners. As LNX1 contains four PDZ domains the authors used a human protein array to identify direct LNX1 PDZ domain binding partners and *Mustn1* was one of 21 out of the original 62 proteins that had carboxy terminal tails that conform to PDZ domain binding motifs, though no direct experimental biochemical verification of a physical interaction between *Mustn1* and LNX1 was provided [61].

Considering all of the current data together, *Mustn1* should be considered, not only as a pan-musculoskeletal cell/tissue marker, but more importantly as a regulatory protein whose expression precedes that of master regulatory genes such as *MyoD* and *Myog* in skeletal muscle and *Sox9* in cartilage (Figure 9). Whether *Mustn1* is part of a multi-protein transcriptional complex responsible for activating these regulatory genes remains experimentally unknown. It is also very interesting to determine whether the same is true for critical transcription factors responsible for osteogenesis and tendogenesis; does *Mustn1* expression in osteoblasts precedes that of *Osx* and *Runx2* and in tenocytes that of *Scx* and *Mkh*? Regardless, its prevalence in key cellular processes such as proliferation and differentiation and more complex tissue-based events as embryonic development, organogenesis and regeneration, leads me to believe that *Mustn1* will become an indispensable and critical early regulatory protein for all major cells of the musculoskeletal system. Hopefully, in the near future additional experimental evidence will emerge to support the suggested regulatory-based hypothesis for this important pan-musculoskeletal gene.

## Figures and Tables

**Table 1:** Summary of *Mustn1* expression in musculoskeletal tissues.

Cell/Tissue	Assay	Observed Expression	Study
**Skeletal Muscle**			
Mouse embryo	In situ Hybridization	Somites, trapezius and intercostal muscles	[17]
Mouse quadriceps	Q-PCR	Embryonic, post-natal and adult development	[17]
Mouse flexor digitorum brevis	Immunohistochemistry	Satellite cells	[15]
Frog embryo	In situ Hybridization	Paraxial mesoderm and somites	[19]
Zebrafish embryo	In situ Hybridization	Segmental plate mesoderm and somites	[12]
Chicken pectoralis major	Microarray/Q-PCR	Post-hatch development (peaked at 6 weeks)	[20]
Chicken breast muscle	RNAseq	One of ten most upregulated genes at 8 weeks	[21]
Chicken pectoralis major and thigh muscle	Q-PCR/Western Blotting/Immunohistochemistry	High expression in both muscles, especially at post-hatch day 49 and beyond in both males and females; peripherally located nuclei of myofibers	[22]
Duck pectoralis major and leg muscle	Q-PCR	High expression in both muscles at 1, 3, 5, 7 and 9 weeks	[23]
Sheep longissimus dorsi	Microarray	Downregulated at 12 weeks	[24]
Pig longissimus dorsi	Microarray/Q-PCR	Downregulated in Korean native vs. Yorkshire pig	[25]
Human quadriceps	Microarray/Q-PCR	Upregulated ~2.9 and ~6-fold at 6 h and 24 h, respectively, during eccentric vs. concentric contractions	[26]
Rat Flexor halucis longus	Q-PCR	Upregulated ~3.7-fold and ~2.4 at 4 and 8 weeks, respectively, of resistance exercise	[27]
Rat Flexor halucis longus	Microarray/Q-PCR	Upregulated ~3-fold and ~1.5 at 4 and 8 weeks, respectively, of resistance exercise	[28]
Rat gastrocnemius and soleus	Microarray	Upregulated ~4-fold only in soleus after a single aerobic exercise bout	[29]
Pig biceps femoris; longissimus dorsi and Vastus intermedius	Microarray/Q-PCR	Upregulated ~2.6-fold only in the Biceps femoris after 3hrs of exercise; in satellite cells of isolated Vastus intermedius	[30]
**Cartilage**			
Rat fracture callus	In situ Hybridization	Proliferating chondrocytes	[5]
Mouse and rat embryo	In situ Hybridization	Mesenchymal condensation of limb buds; perichondrium of vertebral bodies; craniofacial cartilage (branchial arch frontonasal process)	[5,31]
Frog embryo	In situ Hybridization	Mandibular, hyoid, branchial and other head cartilaginous tissues	[19]
Zebrafish embryo	In situ Hybridization	Ceratohyal and ceratobranchial elements of the pharyngeal skeleton	[12]
Rat mandible	Microarray/Q-PCR	Upregulated at days 7 (~2-fold), 9 (~2-fold), 14 (~3-fold) and 21 (~2-fold) during the advancement of the mandibular condyle	[33]
Rat tibial articular cartilage	Q-PCR	Upregulated expression following ultrasound stimulation	[36]
**Bone/Tendon**			
Rat fracture callus	In situ Hybridization	Osteoblasts and osteocytes	[5,38]
Rat fracture callus	Microarray/Q-PCR	Upregulated ~3-fold at post-fracture day 3 in alcohol-fed animals	[39]
Transgenic mice	Microarray	Osteoblasts, preosteocytes and osteocytes	[41]
PTHrP+/+ and −/− mice	Microarray	Upregulated ~2.2-fold in PTHrP+/+ osteoblasts exposed to simulated microgravity and ~3.9-fold in PTHrP−/− osteoblasts at 1 g Osteoblasts	[42]
Rat tendons	Microarray/Q-PCR	Upregulated in in native tendon than in monolayer or the three-dimensional tenocyte cultures	[43]

**Table 2:** Summary of *Mustn1* functional perturbation studies.

Cell/Tissue	Approach	Observed Effects	Study
Myogenic cells (C2C12)	RNAi	Impaired myoblast differentiation, myofusion and myotube formation; Downregulation of myogenic and myofusion marker genes	[17]
Mouse skeletal muscle stem cells	RNAi	Inhibited expansion of skeletal muscle stem cells	[15]
Chondrogenic cells (RCJ3.1C5.18)	RNAi	Reduction in proliferation and differentiation; Downregulation of *Sox9*, *ColII* and *ColIX* mRNA expression	[31]
Frog embryo	Antisense morpholinos	Small or absent eyes, shortened body axis and tail kinks; Downregulation of cranial *Sox9* mRNA expression; Disrupted cartilage formation and in some cases a complete absence of cartilaginous structures associated with the eye, ceratohyal cartilage and gill arches	[19]
Zebrafish embryo	Antisense morpholinos	Curved body axis phenotype; Otolith and left-right asymmetry defects; Curling of cilia and disorganized γ-tubulin expression	[44]
